# High-resolution promoter map of human limbal epithelial cells cultured with keratinocyte growth factor and rho kinase inhibitor

**DOI:** 10.1038/s41598-017-02824-8

**Published:** 2017-06-06

**Authors:** Masahito Yoshihara, Yuzuru Sasamoto, Ryuhei Hayashi, Yuki Ishikawa, Motokazu Tsujikawa, Yoshihide Hayashizaki, Masayoshi Itoh, Hideya Kawaji, Kohji Nishida

**Affiliations:** 1 Department of Ophthalmology, Osaka University Graduate School of Medicine, Suita, Osaka Japan; 2 Division of Genomic Technologies, RIKEN Center for Life Science Technologies, Yokohama, Kanagawa Japan; 3 Department of Stem Cells and Applied Medicine, Osaka University Graduate School of Medicine, Suita, Osaka Japan; 4 RIKEN Preventive Medicine and Diagnosis Innovation Program, Wako, Saitama Japan; 5 Preventive Medicine and Applied Genomics Unit, RIKEN Advanced Center for Computing and Communication, Yokohama, Kanagawa Japan

## Abstract

An *in vitro* model of corneal epithelial cells (CECs) has been developed to study and treat corneal disorders. Nevertheless, conventional CEC culture supplemented with epidermal growth factor (EGF) results in a loss of CEC characteristics. It has recently been reported that limbal epithelial cells (LECs) cultured with keratinocyte growth factor (KGF) and the rho kinase inhibitor Y-27632 could maintain the expression of several CEC-specific markers. However, the molecular mechanism underlying the effect of culture media on LECs remains to be elucidated. To elucidate this mechanism, we performed comprehensive gene expression analysis of human LECs cultured with EGF or KGF/Y-27632, by cap analysis of gene expression (CAGE). Here, we found that LECs cultured with KGF and Y-27632 presented a gene expression profile highly similar to that of CECs *in vivo*. In contrast, LECs cultured with EGF lost the characteristic CEC gene expression profile. We further discovered that CEC-specific *PAX6* promoters are highly activated in LECs cultured with KGF and Y-27632. Our results provide strong evidence that LECs cultured with KGF and Y-27632 would be an improved *in vitro* model in the context of gene expression. These findings will accelerate basic studies of CECs and clinical applications in regenerative medicine.

## Introduction

The corneal epithelium forms the outermost layer of the cornea and acts as a barrier against infection and injury. It consists of stratified corneal epithelial cells (CECs), which turn over every 7–14 days^1^. The corneal epithelium is maintained by limbal epithelial stem cells (LESCs) located in the periphery of the cornea. LESCs at the basal layer of limbal epithelial cells (LECs) divide and give rise to transient amplifying progenitors, which migrate centripetally through the basal layer. These cells finally differentiate into CECs and move vertically through the suprabasal layers^2^. Destruction of LESCs by trauma such as chemical burns or diseases, including Stevens-Johnson syndrome, can result in limbal stem cell deficiency (LSCD), which leads to corneal opacity and vascularization, and consequently severe visual impairment^3^. Corneal transplantation is the most effective method to treat LSCD. However, its availability is limited by a global donation shortage^4^, and it sometimes fails as a result of immunorejection^5^.

To overcome these problems, alternative CECs are differentiated from different sources such as *ex vivo*-expanded limbal epithelial cells (LECs)^6, 7^, oral mucosal epithelial cells^8, 9^, and induced pluripotent stem cell (iPSC)-derived CECs^10–12^. To make these alternative CECs equivalent to CECs *in vivo*, the culture system should be designed to maintain the characteristics of CECs *in vivo*. Epidermal growth factor (EGF) has been widely used to culture CECs because several reports have indicated that EGF stimulates their proliferation and migration via the PI3K/AKT, MAPK/Erk, and NF-κB signaling pathways^13–17^. However, several studies have revealed that EGF suppresses the expression of PAX6^18, 19^, the key regulator of corneal epithelial differentiation and homeostasis^20–23^. This suggests that EGF is not a desirable factor to maintain the characteristics of CECs. In addition to EGF, transforming growth factor alpha (TGFα) and hepatocyte growth factor (HGF) have been reported to inhibit the expression of CEC-specific marker keratin 3 (KRT3)^24^.

Recently, Miyashita *et al*. discovered that LECs cultured with keratinocyte growth factor (KGF) and the rho kinase inhibitor Y-27632 maintained high expression of PAX6 and KRT3 when compared with LECs cultured with EGF, without losing their proliferative ability^25^. However, the molecular mechanism underlying the effect of different culture media on LECs remains poorly understood. To elucidate this mechanism, here we performed a comprehensive gene expression analysis of LECs cultured in these two distinct conditions, by cap analysis of gene expression (CAGE)^26, 27^. This technique enabled us to monitor transcription initiation at the promoter level, which can be interpreted as a quantitative assessment of the regulatory output. Our results provide new insights in the regulation of the characteristic gene expression of CECs and support the development of alternative CECs with better conditions for the treatment of corneal disorders.

## Results

### Culture media drastically altered the gene expression profile of LECs

**Figure 1:**
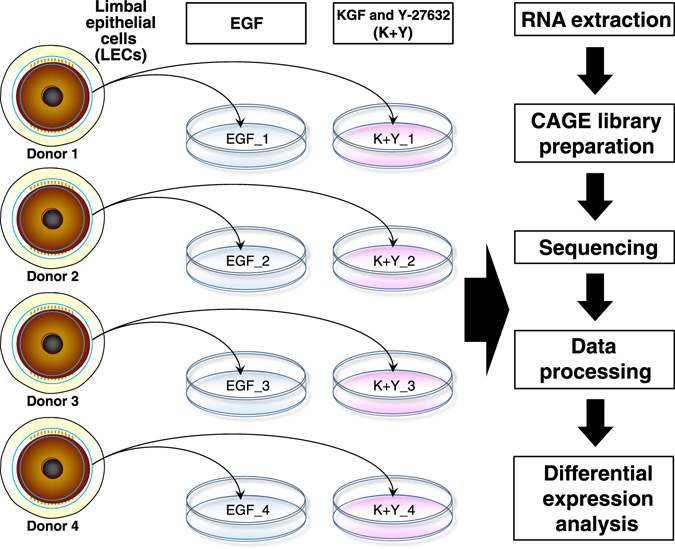
Study design. Distinct limbal epithelial cells (LECs) derived from 4 donors were divided into 2 groups: LECs cultured with EGF (blue) and LECs cultured with KGF and Y-27632 (K+Y, pink). RNA was extracted from LECs and CAGE analysis was performed.

To explore the effect of culture media on LECs, LECs derived from 4 distinct donors were separated and cultured in two different conditions: in the presence of EGF (a conventional method), or in the presence of KGF and Y-27632 (K+Y; a newly developed method). We performed CAGE analysis on these LECs (four cultures with EGF and four with K+Y) to profile them at the promoter level (Fig. 1). We identified 31,406 of 184,827 Functional Annotation of Mammalian Genome (FANTOM) 5-defined promoters^28^ that had >10 CAGE tags in at least one sample. These promoters were further investigated in this study.

To confirm the validity of our study, we first examined the expression level of the CEC-specific markers KRT3 and KRT12 in LECs cultured in the two different conditions. Peaks of the CAGE tags could be observed at the FANTOM5-defined promoter regions of these two genes (p1@KRT3 and p1@KRT12, respectively; Supplementary Fig. S1a). Both of these genes were significantly upregulated in K+Y-treated LECs (adjusted *P* = 3.70 × 10^−24^ and 5.75 × 10^−46^, respectively; Supplementary Table S1). We further confirmed their expression by quantitative reverse transcription PCR (qRT-PCR; Supplementary Fig. S1b). These results were consistent with the previous report^25^, which implies that we were able to reproduce their culture system.

**Figure 2:**
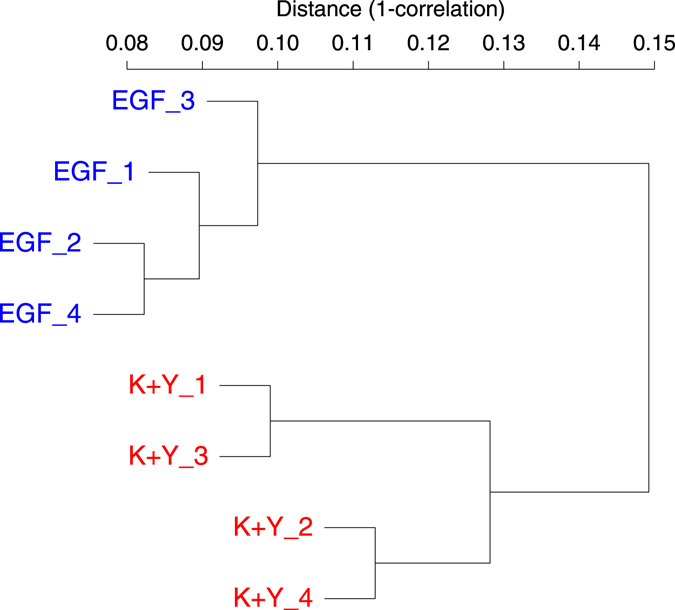
Hierarchical clustering analysis of EGF-treated LECs and K+Y-treated LECs. In total, 31,406 promoters that had more than 10 CAGE tags in at least one sample were examined. Hierarchical clustering analysis was performed using the average linkage algorithm with a Spearman correlation distance matrix. The same numbers correspond to the same donors. LECs in each condition showed a similar expression pattern. K+Y: KGF and Y-27632.

Hierarchical clustering analysis of the eight LEC samples demonstrated that the gene expression pattern was similar in each condition, and the similarity was even greater than that of LEC pairs derived from the same donors (Fig. 2). Principal component analysis (PCA) also showed distinct differences between the two conditions, rather than between different donors (Supplementary Fig. S2). These results indicate that the culture media drastically altered the gene expression profile of LECs.

### Functional analysis of differentially expressed genes across different culture conditions

**Figure 3:**
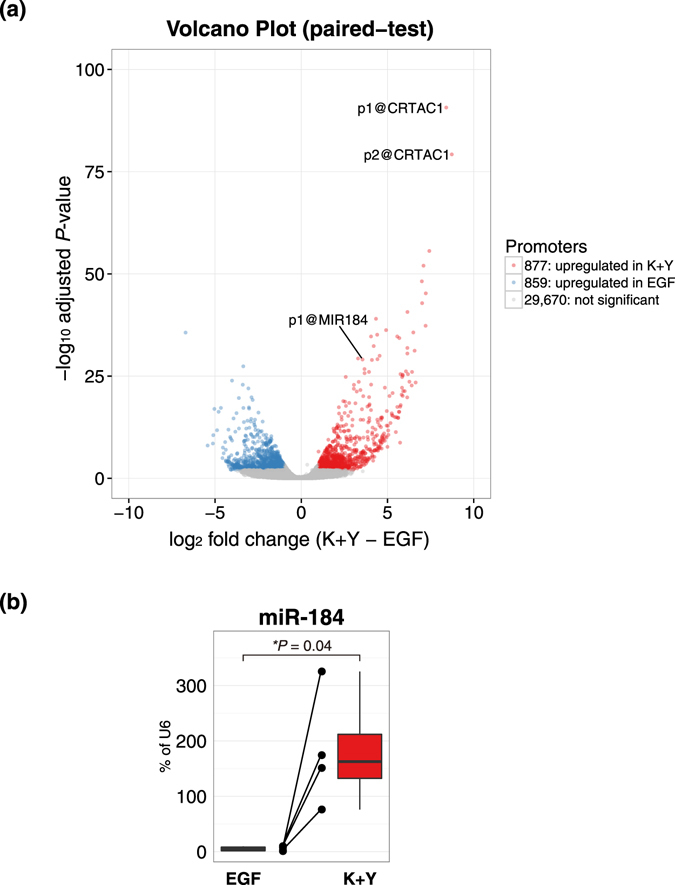
Volcano plot of statistical significance against log 2 fold-change between EGF-treated LECs and K+Y-treated LECs. **(a)** Promoters highly expressed in LECs cultured with EGF are shown in blue (adjusted *P* < 0.01. log 2 fold-change <−1), and promoters highly expressed in LECs cultured with K+Y are shown in red (adjusted *P* < 0.01. log 2 fold-change >1). Promoters that were not differentially expressed between these two culture conditions are shown in gray. The most differentially expressed promoters were p1@CRTAC1 and p2@CRTAC1, which were quite highly expressed in K+Y. The promoter of CEC-specific *miR-184* was also highly expressed in LECs cultured with K+Y. K+Y: KGF and Y-27632. **(b)** Expression level of miR-184 quantified by RT-PCR. Each dot represents the expression level in each sample, and each line indicates binding to samples from the same donors. An asterisk represents statistical significance.

We performed differential expression analysis between the two conditions and found that 877 promoters were significantly upregulated in K+Y-treated LECs and 859 promoters were significantly upregulated in EGF-treated LECs (Fig. 3a and Supplementary Tables S1 and S2). To address how these differentially expressed genes contribute to cellular function, a gene ontology (GO) enrichment analysis was performed using DAVID (http://david.abcc.ncifcrf.gov/). GO enrichment analysis of the K+Y-upregulated genes showed that they were significantly enriched in translational elongation, regulation of cell proliferation, and translation (Table 1). Most genes annotated as translational elongation and translation were ribosomal proteins, possibly because of the acceleration of cell proliferation and protein production^29^. These results are consistent with the previous report indicating that the colony-forming efficiency of LECs was drastically improved by supplementing the medium with K+Y^25^. Moreover, K+Y-upregulated genes were significantly enriched in epidermis and ectoderm development, which suggests that LECs are stably committed towards a CEC lineage. In contrast, GO enrichment analysis of the EGF-upregulated genes showed that they were significantly enriched in immune response, response to wounding, and defense response (Table 2). This is concordant with previous reports showing that EGF stimulates CECs during wound healing^13–15, 17^. Our functional analysis indicates that K+Y medium should be suitable for effective *ex vivo* proliferation of LECs.

**Table 1:** Gene ontology enrichment analysis of genes highly expressed in K+Y-treated LECs.

Term	Count	Genes	*P*-value	FDR
GO:0006414 ~ translational elongation	15	*RPL13AP7, RPS26P8, RPS9, RPL31P49, RPL13AP5, RPL30, RPS28, RPL23, RPS29, RPL32, RPL37A, RPL12, RPL4, RPL10AP6, RPS24*	1.76 × 10^−7^	3.02 × 10^−4^
GO:0008544 ~ epidermis development	17	*PTGS2, PAX6, GJB3, SPINK5, DCT, COL17A1, CDKN2A, KRT27, KRT5, SPRR1A, POU2F3, KRT14, FOXE1, KRT1, DSP, POU3F1, KLF4*	1.59 × 10^−6^	2.74 × 10^−3^
GO:0007398 ~ ectoderm development	17	*PTGS2, PAX6, GJB3, SPINK5, DCT, COL17A1, CDKN2A, KRT27, KRT5, SPRR1A, POU2F3, KRT14, FOXE1, KRT1, DSP, POU3F1, KLF4*	4.44 × 10^−6^	7.63 × 10^−3^
GO:0042127 ~ regulation of cell proliferation	36	*S100A6, FOSL2, PTGS2, IGFBP6, CLU, EGLN3, PAX6, GJA1, KIT, FTH1, S1PR3, GPX1, GPC3, CDKN2A, KRT5, ASPH, THBS1, FGF1, EGFR, RPS9, IGF2, RB1, SPARC, GJB6, GAS1, PLA2G4A, CDKN1A, EPGN, ALOX15B, HBEGF, BMP7, MAB21L1, EMP3, KLF4, TM4SF4, IGFBP5*	1.95 × 10^−5^	3.35 × 10^−2^
GO:0006412 ~ translation	22	*EGFR, RPL13AP7, MRPL3, EIF4BP7, RPS26P8, RPS9, RPL31P49, RPL13AP5, EIF3D, RPL30, RPS28, RPL32, RPS29, RPL23, EIF3E, EIF3L, RPL37A, RPL4, RPL12, MRPL33, RPL10AP6, RPS24*	2.20 × 10^−5^	3.77 × 10^−2^

FDR: false discovery rate < 0.05.

**Table 2:** Gene ontology enrichment analysis of genes highly expressed in EGF-treated LECs.

Term	Count	Genes	*P*-value	FDR
GO:0006955 ~ immune response	62	*PXDN, S100A7, VTCN1, IL19, IFI44L, TNFSF13, IL15, CXCL11, CXCL10, TAPBP, HAMP, CFH, SEMA3C, IL1B, ICAM1, GBP6, BST2, INPPL1, HLA-C, SERPING1, HLA-B, GEM, HLA-F, UNC13D, PPBP, TNFSF13B, CTSC, GBP4, GBP3, GBP2, LCP1, GBP1, IFIH1, YWHAZ, IL1R1, C9, CXCL5, IFITM2, IFITM3, CXCL3, CXCL2, RSAD2, C1R, CXCL6, CD74, IFI35, TAP2, TAP1, FYB, SECTM1, CFB, SAMHD1, TINAGL1, TRIM22, FOXP1, PSMB8, PSMB9, TNFSF10, APOL1, CXCL16, CD14, IFI6*	3.74 × 10^−12^	6.56 × 10^−9^
GO:0009611 ~ response to wounding	48	*YWHAZ, C9, ELF3, S100A8, PDGFB, CXCL3, TNC, CXCL2, S100A9, C1R, CXCL6, IL15, ELK3, CDH3, CXCL11, SRF, MDK, CXCL10, CTGF, SERPINE1, SERPINA3, CFH, IL1B, SERPINA1, SCNN1B, FN1, B4GALT1, KLK6, PLAT, KLF6, KLK8, BMP2, CFB, MAP1B, SERPING1, GRHL3, IDO1, MECOM, APOL2, PLSCR1, UNC13D, TFPI, JAK2, CTSB, ALOX5, HDAC9, PROS1, CD14*	5.21 × 10^−10^	9.13 × 10^−7^
GO:0006952 ~ defense response	50	*S100A8, ELF3, S100A7, S100A9, IL15, CXCL11, TAPBP, CXCL10, HAMP, GATA3, CFH, SERPINA3, IL1B, HCP5, SERPINA1, MX1, SERPING1, HLA-C, HLA-B, MECOM, INHBA, PSG9, UNC13D, PPBP, PSG4, IFIH1, YWHAZ, IL1R1, C9, CXCL3, CXCL2, RSAD2, C1R, CXCL6, ITGB1, CD74, TAP2, TAP1, FN1, B4GALT1, BMP2, CFB, SAMHD1, IDO1, APOL2, APOL1, CXCL16, ALOX5, HDAC9, CD14*	2.19 × 10^−8^	3.83 × 10^−5^
GO:0006954 ~ inflammatory response	28	*YWHAZ, C9, S100A8, ELF3, CXCL3, CXCL2, S100A9, C1R, CXCL6, IL15, CXCL11, CXCL10, CFH, SERPINA3, IL1B, SERPINA1, FN1, B4GALT1, BMP2, CFB, SERPING1, IDO1, MECOM, APOL2, UNC13D, ALOX5, HDAC9, CD14*	9.71 × 10^−6^	1.70 × 10^−2^
GO:0009615 ~ response to virus	15	*IFIH1, BST2, SAMHD1, RSAD2, IFI44, IFI16, STAT1, TRIM22, IFI35, STAT2, PLSCR1, UNC13D, ISG15, XPR1, MX1*	1.31 × 10^−5^	2.28 × 10^−2^
GO:0007565 ~ female pregnancy	15	*MUC1, STS, IDO1, PSG1, PTHLH, APOL2, PSG9, PAPPA, PSG6, PSG5, GRN, CLIC5, PSG4, IL1B, GNAS*	1.45 × 10^−5^	2.55 × 10^−2^
GO:0048002 ~ antigen processing and presentation of peptide antigen	9	*TAP2, IFI30, HLA-C, HLA-B, TAPBPL, CALR, CD74, TAPBP, HLA-F*	2.58 × 10^−5^	4.52 × 10^−2^

FDR: false discovery rate < 0.05.

### K+Y-treated LECs maintain the characteristic gene expression profile of CECs

**Figure 4:**
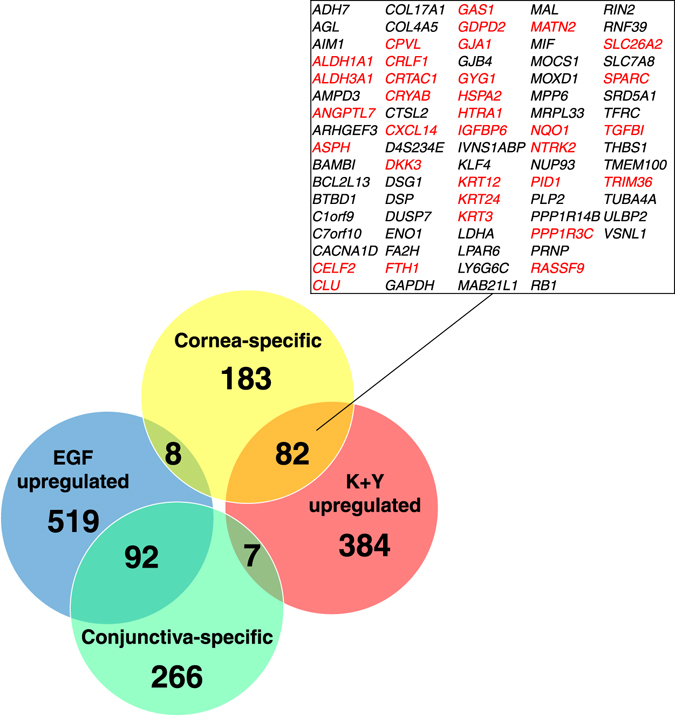
Venn diagram of differentially expressed genes overlapping with cornea and conjunctiva-specific genes. Genes highly expressed in LECs cultured with K+Y are shown in red, and genes highly expressed in LECs cultured with EGF are shown in blue. Genes specifically expressed in the cornea and conjunctiva based on the microarray analysis are shown in yellow and green, respectively. The 82 cornea-specific genes highly expressed in LECs cultured with K+Y are listed, and 33 of them that are considered to be regulated by PAX6 are shown in red characters. K+Y: KGF and Y-27632.

To examine whether CEC-specific genes are upregulated in K+Y-treated LECs at the genome-wide level, we downloaded microarray data for human conjunctival and corneal epithelium (GSE5543^30^) and compared the results with our data. The microarray data showed that 273 genes were specifically expressed in the cornea, and 365 genes were specifically expressed in the conjunctiva. Interestingly, 82 of 273 cornea-specific genes overlapped with genes upregulated in K+Y-treated LECs, whereas only 8 cornea-specific genes overlapped with genes upregulated in EGF-treated LECs (Fig. 4). One of the 82 cornea-specific genes, *CRTAC1* (cartilage acidic protein 1), was most significantly upregulated in K+Y-treated LECs (p1@CRTAC1 and p2@CRTAC1; adjusted *P* = 2.03 × 10^−91^ and 5.94 × 10^−80^, respectively; Fig. 3a and Supplementary Table S1). Another report also indicated that CRTAC1 is abundantly expressed in the human corneal epithelium^31^. As a whole, these 82 cornea-specific genes were significantly differentially upregulated among the K+Y-upregulated genes (Supplementary Fig. S3). In contrast, 92 of 365 conjunctiva-specific genes overlapped with genes upregulated in EGF-treated LECs, whereas only 7 conjunctiva-specific genes overlapped with genes upregulated in K+Y-treated LECs (Fig. 4). These results suggest that the K+Y medium promotes a more proper differentiation of LECs into CECs than EGF.

Interestingly, a promoter of microRNA, p1@MIR184, was significantly upregulated in K+Y-treated LECs (adjusted *P* = 8.77 × 10^−30^; Fig. 3a and Supplementary Table S1). We confirmed its significant upregulation by qRT-PCR (Fig. 3b). It has been reported that miR-184 is essential for corneal epithelial differentiation^32^ and is the most abundant miRNA expressed in the mouse and human cornea^33, 34^. The FANTOM5 atlas also demonstrated that miR-184 is expressed exclusively in ocular tissues (Supplementary Table S3). These observations suggest that miR-184 plays a crucial role in the transcriptional network of CECs and K+Y-treated LECs, given that microRNAs regulate gene expression post-transcriptionally^35^.

### CEC-specific PAX6 promoters are upregulated in K+Y-treated LECs

**Figure 5:**
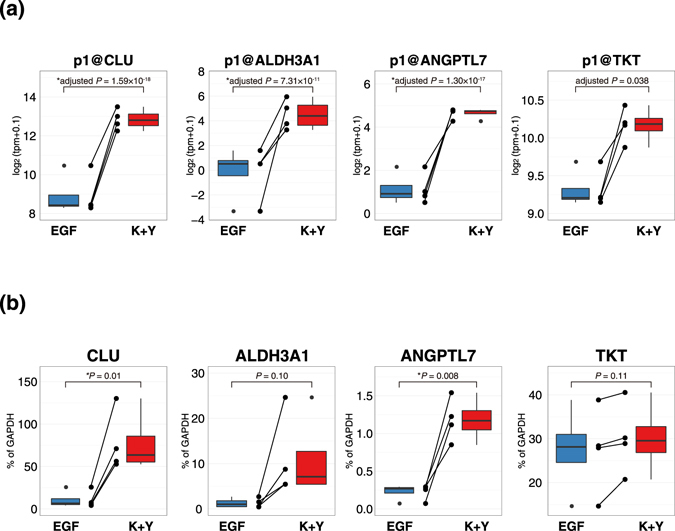
CEC-specific genes regulated by PAX6 are upregulated in K+Y-treated LECs. Expression levels of CEC-specific genes regulated by PAX6 quantified by **(a)** CAGE and **(b)** RT-PCR. Each dot represents the expression levels of distinct promoters or genes in each sample, and each line indicates binding to samples from the same donors. Asterisks represent statistical significance. K+Y: KGF and Y-27632.

A recent study revealed that PAX6 regulates several CEC-specific genes^23^; in particular, clusterin (CLU), aldehyde dehydrogenase 3 family member A1 (ALDH3A1), angiopoietin-like 7 (ANGPTL7) and transketolase (TKT), as well as KRT3 and KRT12 were downregulated in PAX6-depleted human CECs^23^. It should be noted that CLU, ALDH3A1, ANGPTL7, and TKT were also upregulated in K+Y-treated LECs (Fig. 5). Strikingly, 17 of 877 promoters upregulated in K+Y were alternative promoters of *CLU* (Supplementary Table S1), which is one of the most abundant genes expressed in the human CECs and essential for maintaining CECs as non-keratinized^36^. In addition, 33 of 82 (40%) cornea-specific genes upregulated in K+Y-treated LECs were considered to be regulated by PAX6^21^ (Fig. 4). These 33 genes were significantly enriched among both cornea-specific genes and K+Y-upregulated genes (*P* = 3.37 × 10^−10^ and 7.04 × 10^−8^, respectively, Fisher’s exact test). This strongly suggests that PAX6 contributes to the maintenance of CEC characteristics in K+Y-treated LECs as well as CECs *in vivo*.

In K+Y-treated LECs, 22 promoters of 19 transcription factors (TFs) including PAX6, were significantly upregulated (Supplementary Table S1). One of the upregulated TFs, KLF4, has also been reported to be essential for the CEC differentiation. It has been demonstrated that KLF4 regulates the expression of KRT12, ALDH3A1, and TKT^37, 38^. It has also been reported that PAX6 and KLF4 coordinately regulate the CEC-specific genes^22^. These observations suggest that PAX6 and KLF4 co-regulate the expression of CEC-specific genes in K+Y-treated LECs. As an example, there are some putative PAX6 and KLF4 binding motifs in the *KRT12* promoter region, within +/−500 bp around the transcription start site of KRT12 identified by CAGE (Supplementary Fig. S4) as previously reported^38, 39^. Among the other upregulated TFs, DMRTA2, FOXE1, BNC2, ZNF608, and DLX4 were downregulated in the PAX6-knockdown LESCs^21^, suggesting that these TFs are downstream of PAX6. This implies that PAX6 acts as a key regulator of the transcriptional network that controls CEC differentiation in K+Y-treated LECs.

**Figure 6:**
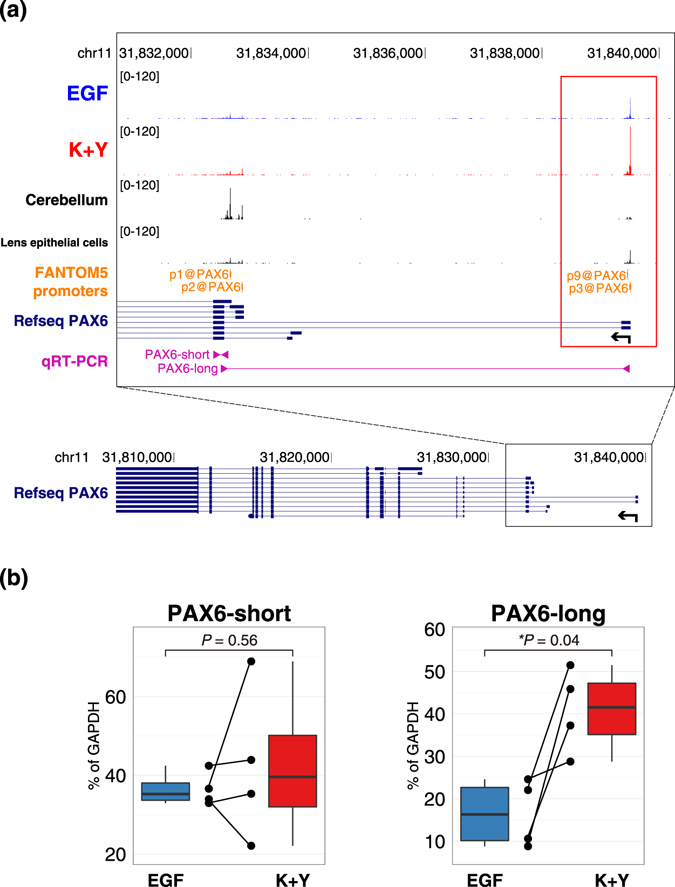
Genome browser view of *PAX6* alternative promoters. **(a)** (Top) EGF represents CAGE tags of LECs cultured with EGF (blue). K+Y represents CAGE tags of LECs cultured with KGF and Y-27632 (red). CAGE tags of the 4 LEC samples cultured in distinct conditions were merged and normalized. p3 and p9@PAX6, indicated by a red rectangle, were highly expressed in K+Y-treated LECs and lens epithelial cells, whereas their expression levels were low in EGF-treated LECs and the cerebellum. p1 and p2@PAX6 were highly expressed in the cerebellum. (Bottom) Zoomed-out view to show the full structure of the *PAX6* gene. Coding exons are represented by thick blocks, whereas untranslated exons are represented by relatively thin blocks. Arrows indicate the direction of transcription. **(b)** Expression levels of two types of *PAX6* transcripts quantified by RT-PCR. The primer positions of each transcript are shown in **(a)**. PAX6-short reflects the activity of p1@PAX6, and PAX6-long reflects the activities of p3 and p9@PAX6. Each dot represents the expression level in each sample, and each line indicates binding to samples from the same donors. An asterisk represents statistical significance.

Interestingly, p3 and p9@PAX6 were remarkably upregulated in K+Y-treated LECs, whereas other alternative promoters, including the main promoters, p1 and p2@PAX6, were not differentially activated (Fig. 6a and Supplementary Fig. S5). These two promoters, p3 and p9@PAX6, are located approximately 7 kb upstream of the other promoters (Fig. 6a). To validate their alternative promoter usage, we designed two types of *PAX6* primers (Fig. 6a). PAX6-short, which reflects the activity of p1@PAX6, was not differentially expressed between the two conditions. In contrast, PAX6-long, which reflects the activities of p3 and p9@PAX6, was significantly upregulated in K+Y-treated LECs (Fig. 6b). The FANTOM5 atlas demonstrated that p3 and p9@PAX6 are exclusively expressed in ocular tissues including lens and CECs, whereas p1 and p2@PAX6 are highly expressed in nervous tissues such as the cerebellum and neural stem cells (Fig. 6a and Supplementary Table S4). These findings indicate that K+Y-treated LECs maintain the characteristic gene expression profile of CECs through activation of CEC-specific *PAX6* promoters.

## Discussion

To realize regenerative medicine and drug discovery, it is necessary to develop *ex vivo* CECs that maintain the characteristics of CECs *in vivo* as an alternative to CECs *in vivo*. A previous study demonstrated that KGF secreted from limbal fibroblasts^40^ did not affect the expression of the CEC-specific marker KRT3, whereas EGF inhibited expression of KRT3 in cultured human CECs^24^. Furthermore, KGF has been reported to promote *ex vivo* proliferation of LECs via the p38 pathway^41^. The rho kinase inhibitor Y-27632 is known to increase the colony-forming efficiency of various cell types, including LECs, by improving their adherence and reactive oxygen species scavenging capacity^42–44^. Recently, Miyashita *et al*. developed a novel culture system supplemented with KGF and Y-27632, and demonstrated by immunohistochemical staining that LECs cultured in this system could maintain high protein expression of PAX6 and several CEC-specific markers such as KRT3 and KRT12^25^. They also found that the colony-forming efficiency of these LECs was much higher than that of LECs supplemented with EGF^25^. To investigate how gene expression of LECs varies across different culture systems, we followed this system and performed a comprehensive promoter analysis of LECs cultured in two distinct conditions: in the presence of EGF, and in the presence of KGF and Y-27632. Here, we demonstrated that LECs supplemented with KGF and Y-27632 shared a highly similar gene expression pattern with CECs *in vivo*, which indicates that these LECs appropriately differentiated into CECs. We further confirmed that KGF facilitates the *ex vivo* proliferation of LECs in a concentration-dependent manner, reaching a maximum at 20 ng/ml which was used in this study (Supplementary Fig. S6). Our findings demonstrate that LECs cultured with KGF and Y-27632 are desirable as substitutes for CECs *in vivo*.

In contrast, EGF-treated LECs showed a conjunctiva-like gene expression pattern. Although the activity levels of the major promoters defined by p1 and p2@PAX6 were similar in different conditions, the CEC-specific promoters located approximately 7 kb upstream to the major promoters, *i.e*., p3 and p9@PAX6, were significantly downregulated in EGF-treated LECs. Furthermore, various CEC-specific genes were downregulated in EGF-treated LECs, and many of these genes have been reported to be regulated by PAX6. It should be noted that these alternative promoters do not contribute to alternative protein isoforms (Fig. 6a bottom). These findings indicate that the upregulation of p3 and p9@PAX6 markedly promotes PAX6 expression, which contributes to the differentiation of LECs into CECs and the maintenance of the CEC-specific gene expression profile when the cells are cultured with KGF and Y-27632. It is also possible that the low expression of PAX6 in EGF-treated LECs cannot facilitate the proper differentiation of LECs, leading to acquisition of conjunctiva-like characteristics.

EGF suppresses the expression of PAX6 in CECs via CCCTC binding factor (CTCF)^18, 19^. CTCF acts as an insulator that blocks the interaction between enhancer and promoter^45^. CTCF binds upstream of the cornea-specific *Pax6* promoter and represses the effect of the ectoderm enhancer in mice^46^. These observations support our findings that the CEC-specific promoters p3 and p9@PAX6 were downregulated in LECs cultured with EGF. In LECs cultured with KGF and Y-27632, CTCF’s binding upstream of these promoters might be hindered by DNA methylation because CTCF-binding sites are generally sensitive to DNA methylation^47, 48^. Effects of culture conditions on epigenetic changes should also be elucidated.

MicroRNAs are known to regulate the expression of other genes by binding to the 3′-untranslated regions (UTR) of target mRNAs^35^. A point mutation in miR-184 causes corneal endothelial dystrophy, iris hypoplasia, congenital cataract, and stromal thinning (EDICT) syndrome, which exhibits a phenotype similar to that of anterior segment dysgenesis disorders caused by PAX6 deficiency^49^. Knockdown of miR-184 leads to decreased expression of PAX6 and KRT3 in human embryonic stem cell-derived CECs^32^, which indicates that miR-184 regulates the expression of PAX6 in CECs. Here, we determined that miR-184 and CEC-specific p3 and p9@PAX6 were significantly upregulated in LECs cultured with KGF and Y-27632. These observations suggest that these CEC-specific *PAX6* promoters are positively regulated by miR-184. It has been reported that the 3′-UTR of PAX6 does not have predictable binding sites for miR-184, which suggests that miR-184 regulates PAX6 indirectly^32^. It is possible that miR-205, which was also upregulated in K+Y-treated LECs (Supplementary Table S1), is involved in this regulation because miR-184 inhibits the binding of miR-205 to its target mRNA and prevents the knockdown effect by miR-205, thereby rescuing the production of target mRNAs^50^. The expression of PAX6 might be rescued by miR-184 through this mechanism. It is also possible that miR-184 inhibits the translation of CTCF, considering that CTCF expression was not significantly different between the two conditions, at least at the transcription level (Supplementary Fig. S7). Further studies are needed to elucidate the underlying mechanism of gene regulation by miR-184.

In summary, we performed CAGE analysis of LECs cultured in two distinct conditions and demonstrated that LECs supplemented with KGF and Y-27632 shared a highly similar gene expression pattern with CECs *in vivo*. Furthermore, we discovered that CEC-specific *PAX6* promoters are highly upregulated in LECs supplemented with KGF and Y-27632, which should be the key regulators of CEC differentiation and maintenance. We recently succeeded in inducing CECs from iPSCs cultured with KGF and Y-27632^11, 12^. Our findings provide strong evidence that LECs cultured with KGF and Y-27632 represent a better *in vitro* model for basic studies of CECs and could be widely utilized for translational research and clinical applications in regenerative medicine.

## Methods

### Culture of human LECs

All human samples were handled according to the tenets of the Declaration of Helsinki. Four research-grade corneas from 4 cadaver human donors were obtained from Sight Life (Seattle, WA, USA), where two donors per sex, aged under 70 years, were chosen. Donor information is presented in Supplementary Table S5. After the endothelium and Descemet’s membrane were peeled away, the central cornea was punched out by using an 8.0-mm-diameter trephine. Limbal parts were treated with dispase II (Thermo Fisher Scientific, Waltham, MA, USA) for 1 h at 37 °C, and the limbal epithelium was scraped from the stroma and dissociated using TrypLE Express (Thermo Fisher Scientific) for 20 min at 37 °C. LECs from each donor were separately cultured in the following two conditions for 17–19 days (10 days after the cells reached 80% confluency) in 5% CO_2_ at 37 °C: 1) CnT-20 (CELLnTEC, Bern, Switzerland) medium, which includes 10 ng/mL EGF, followed by Dulbecco’s Modified Eagle Medium (DMEM): F12 Medium (1:1) (Thermo Fisher Scientific) supplemented with 2% B-27 supplement (Thermo Fisher Scientific) and 10 ng/mL EGF (R&D Systems, Minneapolis, MN, USA) after subconfluency, and 2) DMEM:F12 Medium supplemented with 2% B-27 supplement, 20 ng/mL recombinant human KGF/FGF-7 (R&D Systems), and 10 μM Y-27632 (Wako Pure Chemical Industries, Osaka, Japan). The cells were cultured on dishes coated with the E8 fragment of laminin 511 at 0.5 μg/cm^2^ (i-Matrix-511, Nippi, Tokyo, Japan)^25^.

### RNA preparation

After 17–19 days of culture, LECs were harvested and lysed with 700 μL of QIAzol Lysis Reagent (QIAGEN Inc., Valencia, CA, USA). Total RNA was extracted by Qiagen miRNeasy Mini Kit (QIAGEN Inc.) according to the manufacturer’s protocol. The extracted RNA was quantified using a Nanodrop spectrophotometer (Thermo Fisher Scientific), and qualified using an Agilent BioAnalyzer 2100 (Agilent Technologies, Santa Clara, CA, USA). The RNA integrity number (RIN) of each sample is presented in Supplementary Table S5.

### CAGE library preparation

CAGE libraries were prepared from total RNA as previously described^27^. Briefly, 5 μg of total RNA from each sample was subjected to reverse transcription using SuperScript III Reverse Transcriptase (Life Technologies, Carlsbad, CA, USA) with random primers, and the diols at the 5′ and 3′ ends of the double stranded-RNA/cDNA hybrids were then oxidized with NaIO_4_. The oxidized dialdehyde derivatives were then biotinylated with biotin hydrazide (Vector Laboratories, Burlingame, CA, USA). After the remaining single-stranded RNAs were digested with RNase I (Promega, Madison, WI, USA), the biotinylated 5′-end cap structure of RNA/cDNA hybrids was captured with streptavidin-coated magnetic beads (Dynabeads M-270 Streptavidin; Life Technologies). The single-stranded cDNAs were released from RNAs by heat denaturation, followed by purification with the Agencourt AMPure XP Kit (Beckman Coulter, Brea, CA, USA). After ligation of 5′ adaptors containing barcoded sequences and 3′ adaptors with Illumina adaptor sequences, second-strand cDNAs were synthesized with DeepVent (exo−) DNA polymerase (New England BioLabs, Ipswich, MA, USA). The double-stranded cDNAs were then treated with exonuclease I (New England BioLabs) to remove the extra adaptors and purified using the Agencourt AMPure XP Kit. The resulting CAGE libraries were sequenced using single-end reads of 50 bp on the Illumina HiSeq 2000 (Illumina, San Diego, CA, USA). A summary of sequence statistics is presented in Supplementary Table S5. All CAGE sequence data analyzed in this study have been deposited to the DDBJ Sequence Read Archive (http://trace.ddbj.nig.ac.jp/dra/index_e.html) under accession number DRA005293.

### Annotation of promoters and differential expression analysis

After base calling, artificial sequences were removed using TagDust^51^, and ribosomal RNA sequences were removed with rRNAdust^52^. The resulting extracted CAGE tags were then mapped to the human genome (hg19) using BWA v0.5.9^53^ with the MOIRAI pipeline platform^52^. Mapped CAGE tags were counted with respect to the FANTOM5-defined CAGE peaks, which corresponded to promoters^28^. When multiple CAGE peaks were associated with a single gene, distinct numbers were assigned to individual peaks to distinguish one from the others. For example, p1@PAX6 and p2@PAX6 are distinct CAGE peaks and both of them are associated with the PAX6 gene. CAGE peaks that had fewer than 10 CAGE tags in all 8 samples were excluded. Raw tag counts were normalized against the total tag counts per sample, and then normalized tags per uniquely mapped million tags (tpm) were calculated using the relative log expression (RLE) method. Hierarchical clustering analysis was performed using the average linkage algorithm with a Spearman correlation distance matrix. Differential expression analysis was performed using the Bioconductor package ‘edgeR’ version 3.10.2^54^. Differentially expressed promoters were defined as promoters different with Benjamini-Hochberg-adjusted *P* < 0.01 along with a mean fold change >2 between paired samples derived from corresponding donors. The biological functions of the differentially expressed genes were examined using the GO enrichment analysis tool in DAVID (http://david.abcc.ncifcrf.gov/). GO terms with a false discovery rate (FDR) < 0.05 were defined as significantly enriched.

### Quantitative reverse transcription PCR (qRT-PCR)

Total RNA extracted from the 8 LEC samples was inputted into reverse transcription PCR with the SuperScript III First-Strand Synthesis System (Life Technologies), and miRNA was reverse-transcribed using the Taqman MicroRNA Reverse Transcription Kit (Thermo Fisher Scientific) according to the manufacturers’ instructions. The resulting cDNA was used as a template for quantitative PCR using the ABI Prism 7500 Fast Sequence Detection System (Applied Biosystems, Foster City, CA). TaqMan Gene Expression Assay probes (Supplementary Table S6) and Taqman Fast Universal PCR Master Mix (Thermo Fisher Scientific) were used, and expression values were normalized to GAPDH expression levels as an internal control. The thermocycling program was performed as follows: an initial cycle at 95 °C for 20 sec, followed by 45 cycles of 95 °C for 3 sec and 60 °C for 30 sec. For quantification of miRNA levels, TaqMan MicroRNA Assay probes (Supplementary Table S7) and Taqman Fast Advanced Master Mix (Thermo Fisher Scientific) were used, and expression values were normalized to those of U6 snRNA as an internal control. The thermocycling program was performed as follows: an initial cycle at 50 °C for 2 min and 95 °C for 20 sec, followed by 45 cycles of 95 °C for 3 sec and 60 °C for 30 sec. Additionally, we designed two types of *PAX6* primers (PAX6-long and PAX6-short) to distinguish the expression of the alternative *PAX6* promoters (Fig. 6a and Supplementary Table S8). For the quantification of these promoters, SYBR Premix Ex Taq GC (Perfect Real Time) (TaKaRa Bio, Otsu, Japan) was used, and expression values were normalized to those of GAPDH as an internal control. The thermocycling program was performed as follows: an initial cycle at 95 °C for 30 sec, followed by 45 cycles of 95 °C for 10 sec and 60 °C for 30 sec. *P*-values were calculated using the paired t-test between samples derived from the same donors. *P* < 0.05 was defined as statistically significant.

### Cell proliferation assay

A research-grade cornea from a 69-year-old female (Sight Life) was processed and LECs were cultured with KGF and Y-27632 as described above, except that the concentration of KGF was varied among 0, 2.5, 5, 10, 20, 40, and 100 ng/ml. After 6 days of culture, cell viability was assessed using AlamarBlue Cell Viability Reagent (Thermo Fisher Scientific) before the cells reached confluency. The fluorescence intensity was measured using a microplate reader (2030 ARVO X4, PerkinElmer, Boston, MA, USA). Phase-contrast microscopic images were obtained using an Axio-observer D1 microscope (Carl Zeiss, Jena, Germany) at day 19.

### Microarray data processing

The microarray data were downloaded from the Gene Expression Omnibus (GEO) database^55^ under the accession number GSE5543^30^. In this dataset, human conjunctival and corneal epithelia were analyzed in three independent experiments. Differentially expressed genes were identified using the significance analysis of microarrays (SAM)^56^ according to the weighted average difference method (WAD)^57^. The differentially expressed genes were defined as genes with |SAM score × weight| >1.

### RNA-seq data processing

The RNA-seq data were downloaded from the GEO database^55^ under the accession number GSE54322^21^, which includes CECs and LESCs after knock-down of PAX6. The quality of the RNA-seq reads was evaluated using FastQC (http://www.bioinformatics.babraham.ac.uk/projects/fastqc/), and the processed reads were aligned to the reference genome hg19 using Tophat (version 2.0.9)^58^. The reads mapped to each gene were counted by HTSeq v0.5.4p3^59^. Genes with fewer than 10 reads in all four samples were excluded. The read counts were normalized using the RLE method, and differentially expressed genes were identified using the BioConductor ‘edgeR’ software package^54^ based on Benjamini-Hochberg-adjusted *P* < 0.01 along with a mean fold change >2 between the two types of samples.

### FANTOM5 database

All data of FANTOM5-defined promoters and the expression table were obtained from http://fantom.gsc.riken.jp/5/.

### MOTIF analysis

The potential transcription factor-binding motifs within the *KRT12* promoter region were identified using the FIMO tool from the MEME suite^60^ and the JASPAR CORE database^61^ with *P* < 0.005.

## Electronic supplementary material

Supplementary Information

Supplementary Table S1

Supplementary Table S2
