# Supplementary material for: High-resolution promoter map of human limbal epithelial cells cultured with keratinocyte growth factor and rho kinase inhibitor
Source: Sci Rep. 2017 Jun 6;7:2845. doi: 10.1038/s41598-017-02824-8 (PMC5460231; doi:10.1038/s41598-017-02824-8)
Supplement: Supplementary file 1 — Supplementary Information [file 41598_2017_2824_MOESM1_ESM.pdf]

Supplementary Information

High-resolution promoter map of human limbal epithelial cells cultured with keratinocyte growth factor and rho kinase inhibitor

Masahito Yoshihara, Yuzuru Sasamoto, Ryuhei Hayashi, Yuki Ishikawa, Motokazu Tsujikawa, Yoshihide Hayashizaki, Masayoshi Itoh, Hideya Kawaji, Kohji Nishida

Supplementary Figure S1

(a)

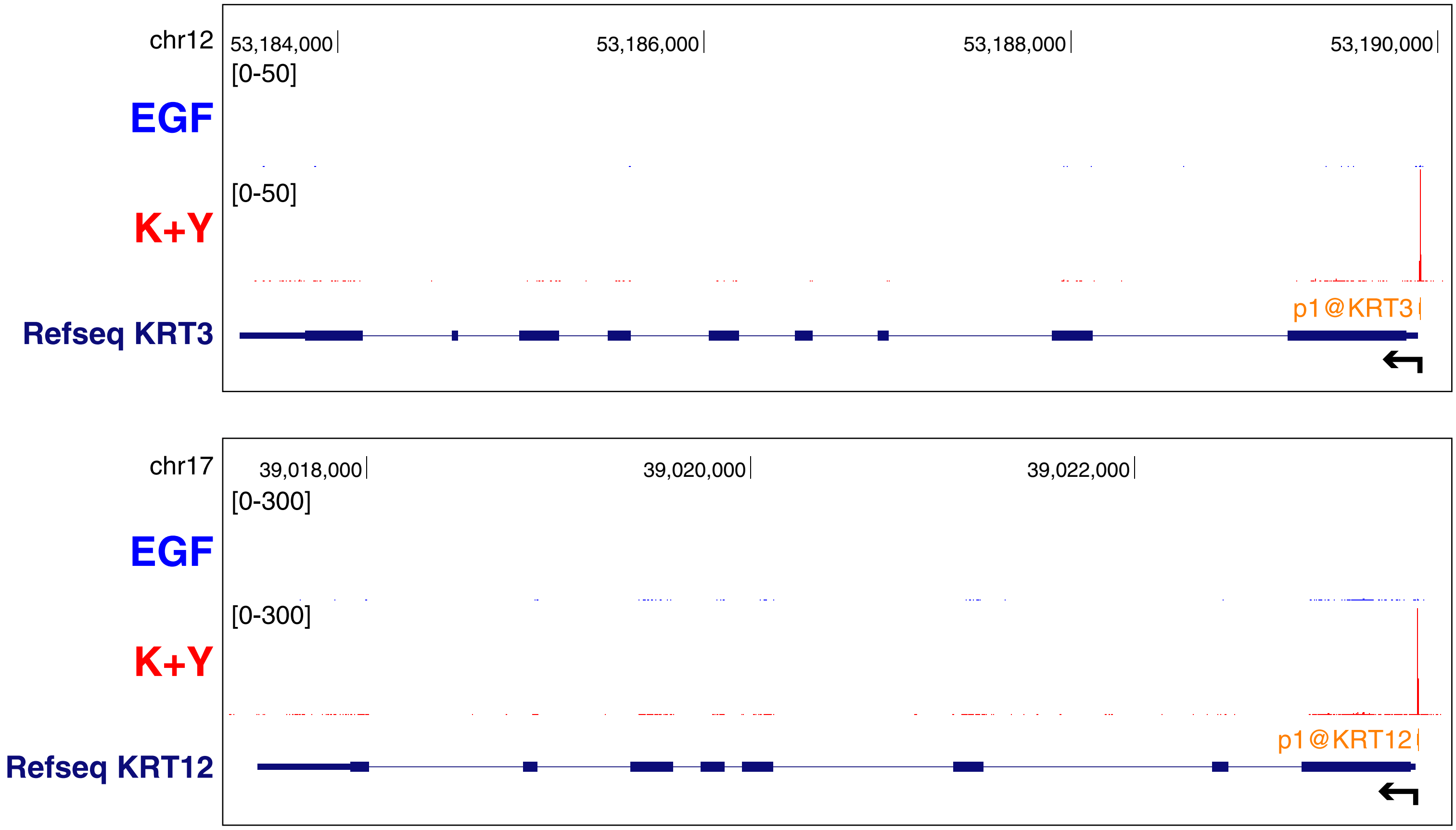

(b)

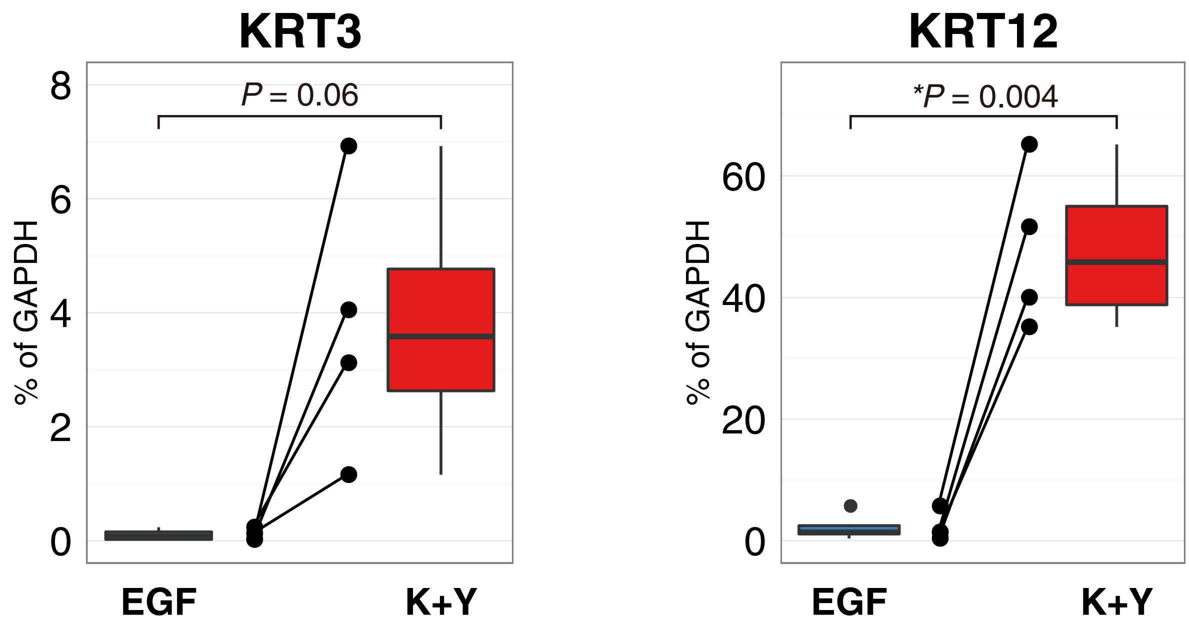

Supplementary Figure S1. Expression level of *KRT3* and *KRT12* promoters identified by CAGE.

(a) EGF represents CAGE tags of LECs cultured with EGF (blue). K+Y represents CAGE tags of LECs cultured with KGF and Y-27632 (red). CAGE tags of the 4 LEC samples cultured in distinct conditions were merged and normalized. Peaks of the CAGE tags could be observed at the p1@KRT3 (top) and p1@KRT12 (bottom) in K+Y. Arrows indicate the direction of transcription. **(b)** Expression levels of KRT3 and KRT12 quantified by RT-PCR. Each dot represents the expression levels of distinct genes in each sample, and each line indicates binding to samples from the same donors. An asterisk represents statistical significance.

Supplementary Figure S2

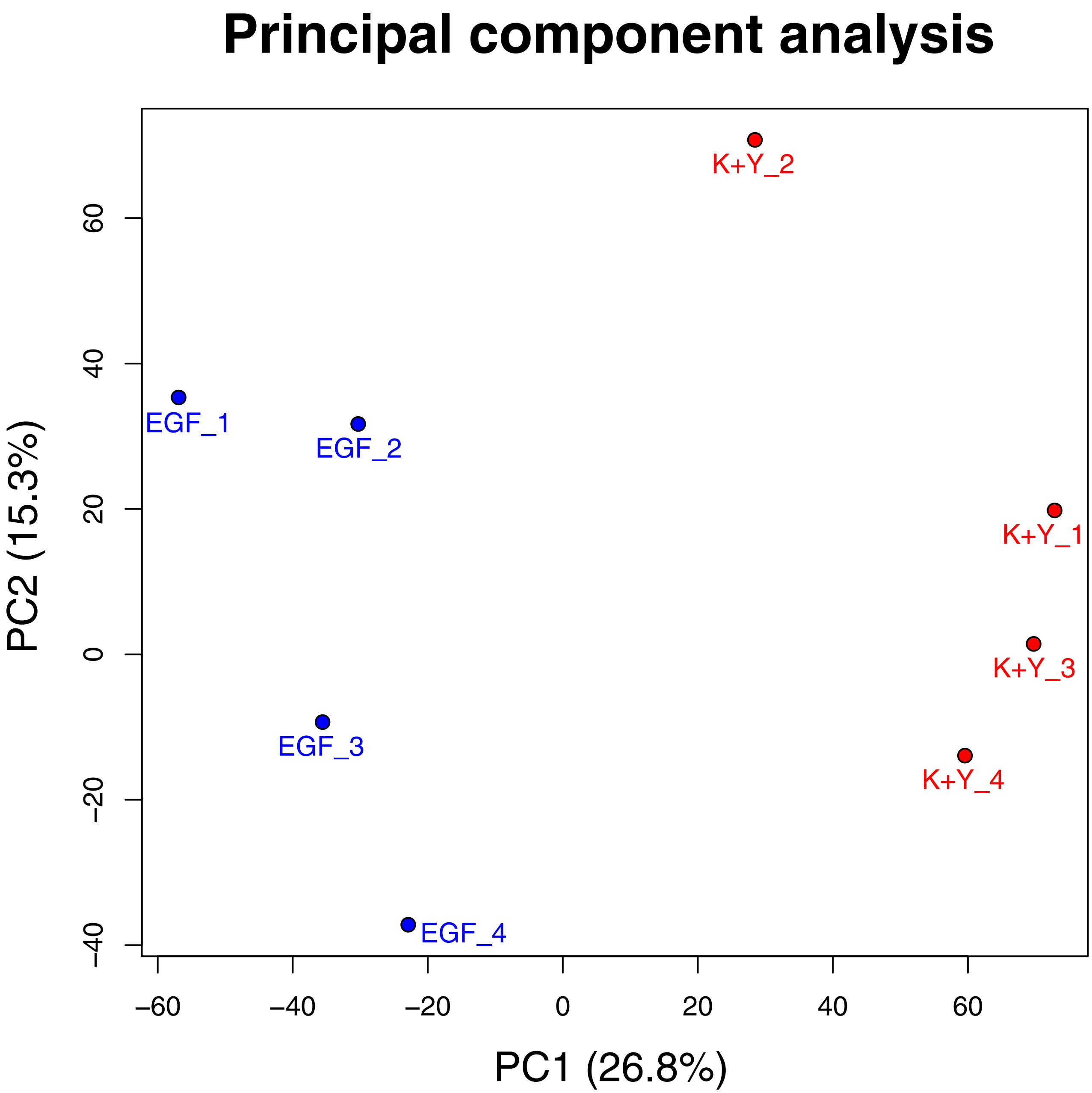

Supplementary Figure S2. Principal component analysis (PCA) of EGF-treated LECs (blue) and K+Y-treated LECs (red).

The same numbers correspond to the same donors. K+Y: KGF and Y-27632.

Supplementary Figure S3

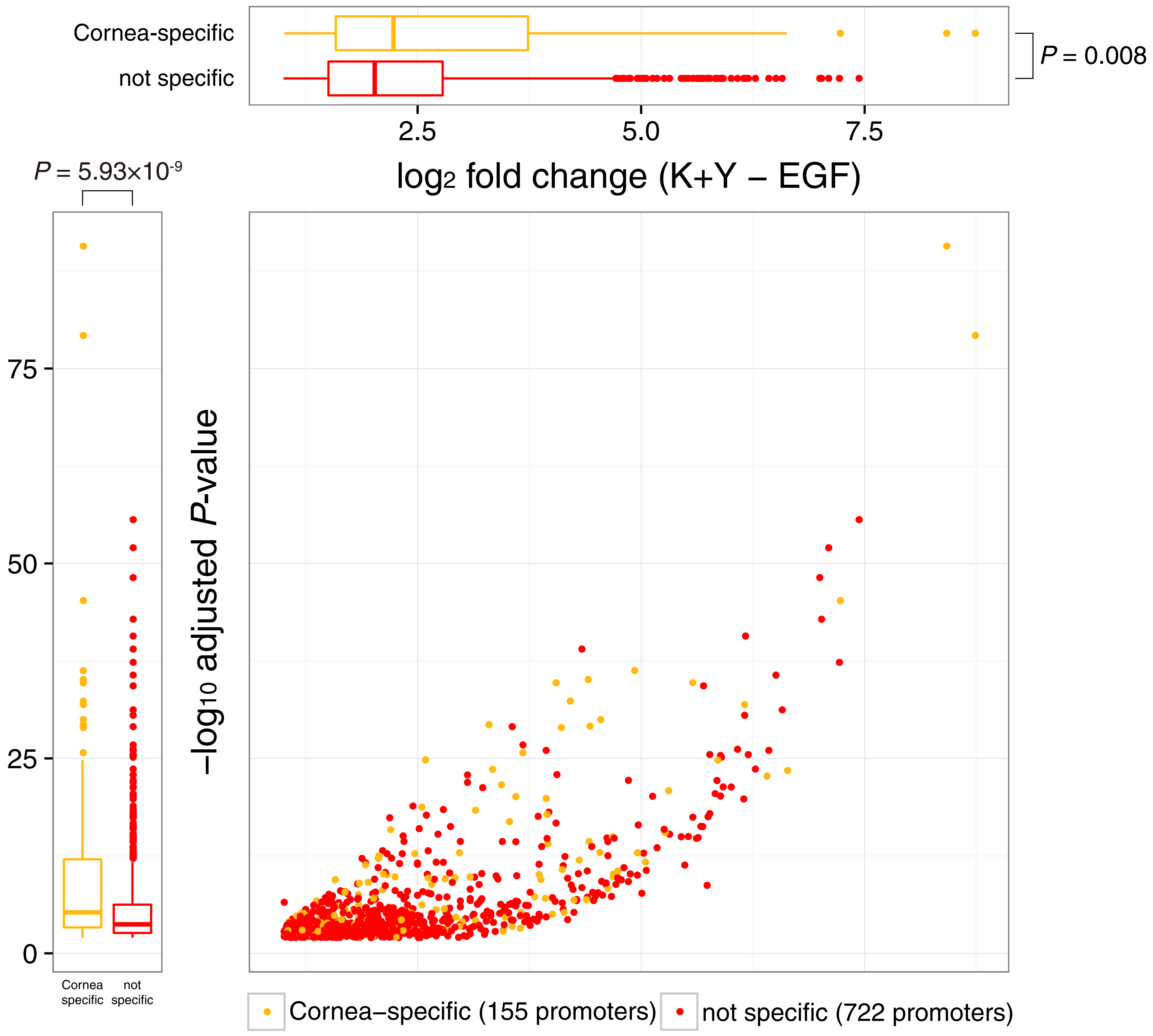

Supplementary Figure S3. Volcano plot of 877 promoters highly expressed in K+Y-treated LECs.

In total, 155 promoters of 82 cornea-specific genes are shown in orange, and the other 722 promoters are shown in red. (Top) Boxplot of log 2 fold-change between EGF-treated LECs and K+Y-treated LECs. Promoters of cornea-specific genes are significantly more upregulated than the other promoters ($P = 0.008$, Wilcoxon rank sum test). (Left) Boxplot of -log 10-transformed adjusted *P*-value. Promoters of cornea-specific genes are significantly differentially upregulated than the other promoters ($P = 5.93 \times 10^{-9}$, Wilcoxon rank sum test).

Supplementary Figure S4

(a)

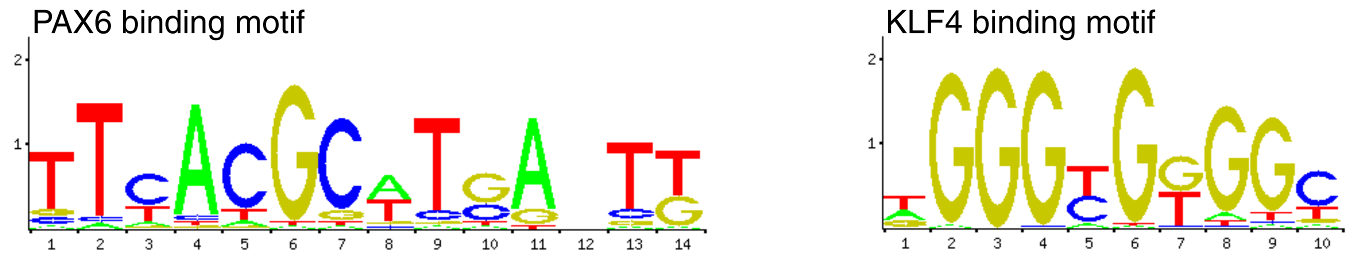

(b)

−500 GAAGCTGTATTCCACTAAATTAACGTATCCATTAGGTATACTTGATCATGACTGGATTTTACATTGGATTGGTTTTTCCAAAGGAAACAAATGGTTTACA

−400 CTGGATTTTCCAAAGGAAACAAACAGAAAAGATCAGGGAAGAACTTAAAAAAGCATAAGGTTTAGGAAGAAGTATCAGCAATCGCTCATTATCTTCCTAC

−300 TGTGAACAAGACGCTAGGCAGTGCTGGGAATACAGGCAACAGACTAATTTGGAGAAGAAAAATTCCTTATGACAAGCAGACTCTACTCCACCCAGCTTTC

−200 TTTTTCATATAACAAATATTTAATGGAAATGATTATTCGATTATTTCTGTATCAATGAATTTTCATTATACATAAATCATTCACTTGTTGAGCTGGCCAA

−100 AAACCTGGGGGGAGGATCCAATTTTGAGTGGAGAAAGTGAACTTTTCAACTGCGACACCCATCTTGCAGCCTATATAAGTTTAGCTTTCTGGCTTGCTGG

0 CACAACTTCCTCTCCAGTTGTGGCCACCTTCCCCAGGCCATGGATCTCTCCAACAACACCATGTCACTCTCAGTGCGCACCCCCGGACTGTCCCGGCGGC

p1@KRT12

+100 TCTCCTCGCAGAGTGTGATAGGCAGACCCAGGGGCATGTCTGCTTCCAGTGTTGGAAGTGGTTATGGGGGAAGTGCCTTTGGCTTTGGAGCCAGCTGTGG

+200 GGGAGGCTTTTCTGCTGCTTCCATGTTTGGTTCTAGTTCCGGCTTTGGGGGTGGCTCCGGAAGTTCCATGGCAGGAGGACTGGGTGCTGGTTATGGGAGA

+300 GCCCTGGGTGGAGGTAGCTTTGGAGGGCTGGGGATGGGATTTGGGGGCAGCCCAGGAGGTGGCTCTCTAGGTATTCTCTCGGGCAATGATGGAGGCCTTC

+400 TTTCTGGATCAGAAAAAGAAACTATGCAAAATCTTAATGATAGATTAGCTTCCTACCTGGATAAGGTGCGAGCTCTAGAAGAGGCTAATACTGAGCTAGA

Supplementary Figure S4. PAX6 and KLF4 binding sites in the *KRT12* promoter region.

(a) PAX6 and KLF4 binding motifs. **(b)** Putative PAX6 (highlighted in red) and KLF4 (highlighted in blue) binding motifs in the *KRT12* promoter region identified by CAGE.

Supplementary Figure S5

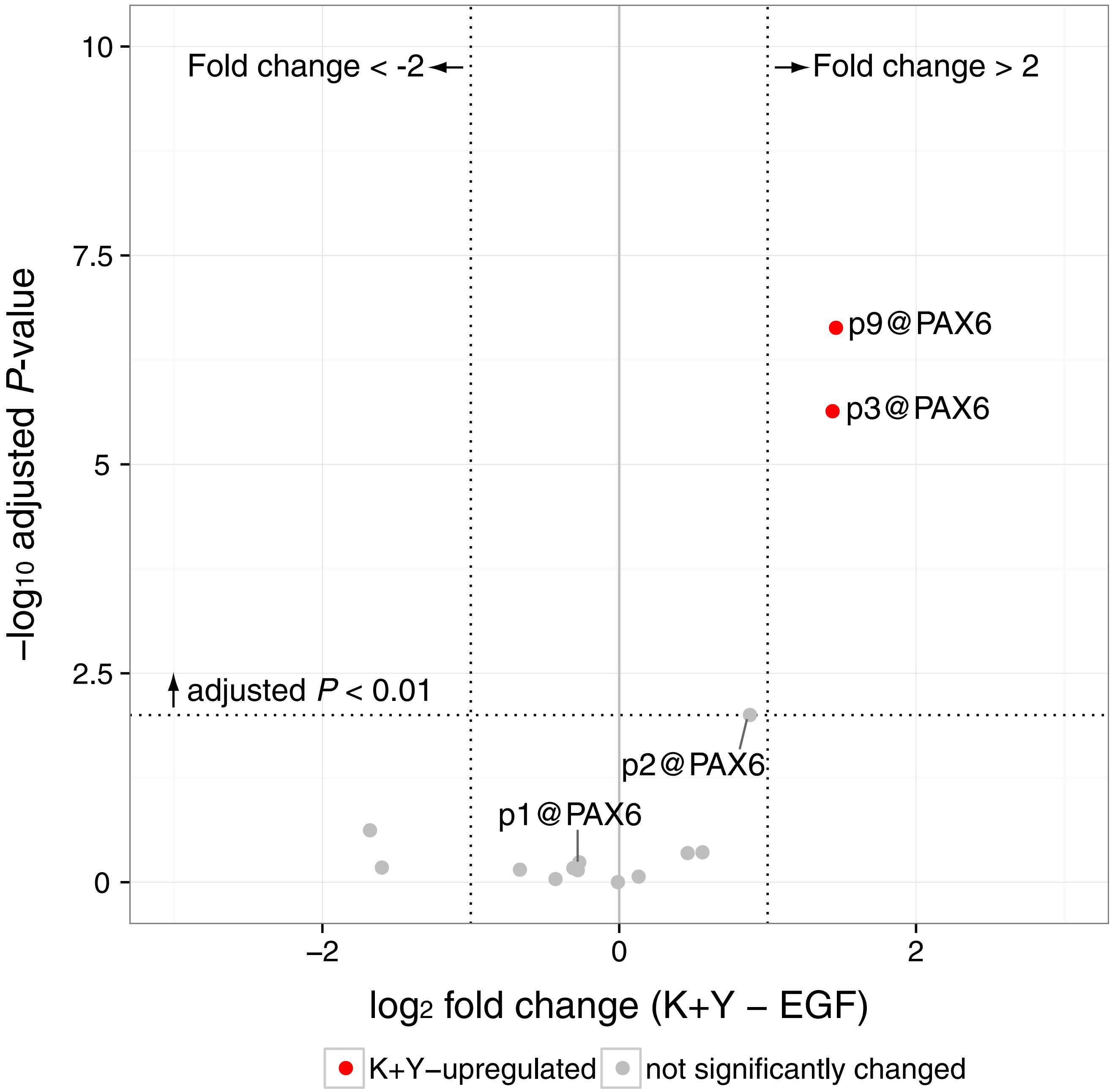

Supplementary Figure S5. Expression changes of the alternative promoters of *PAX6* between the two conditions.

Each dot represents a total of 12 alternative promoters of *PAX6*. Differentially expressed promoters (p3 and p9@PAX6) are shown in red. Dotted lines indicate the thresholds for differentially expressed promoters in this study.

Supplementary Figure S6

(a)

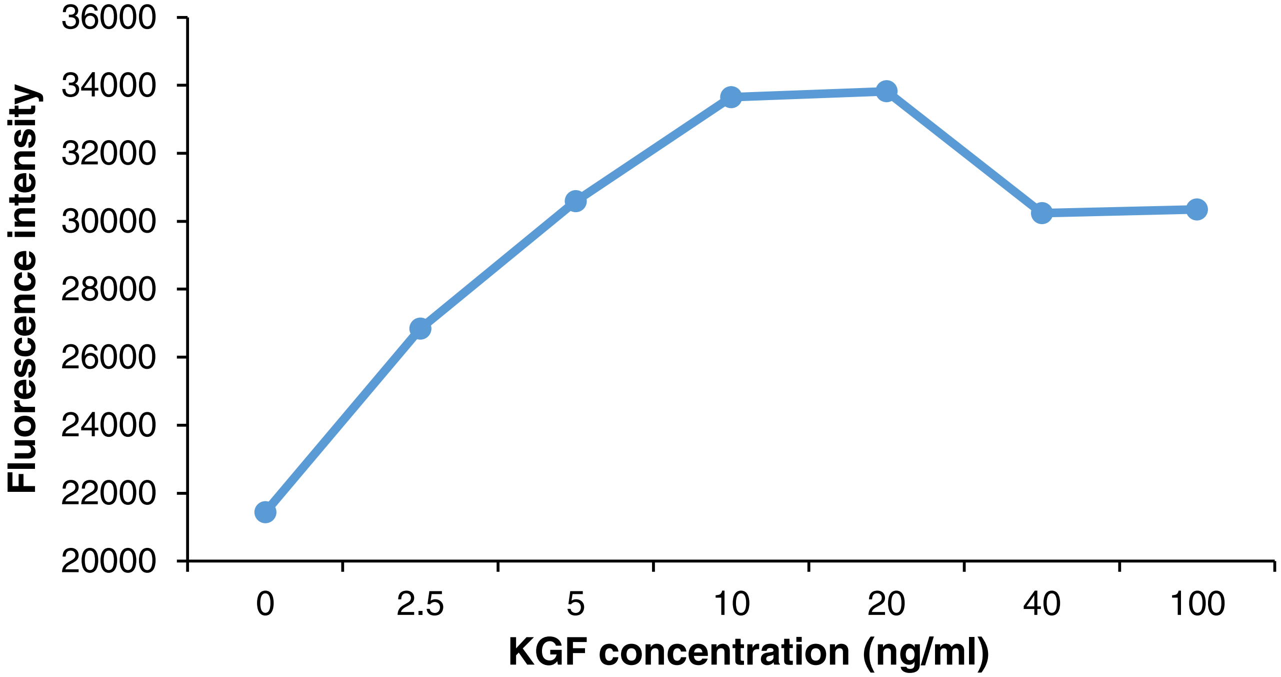

(b)

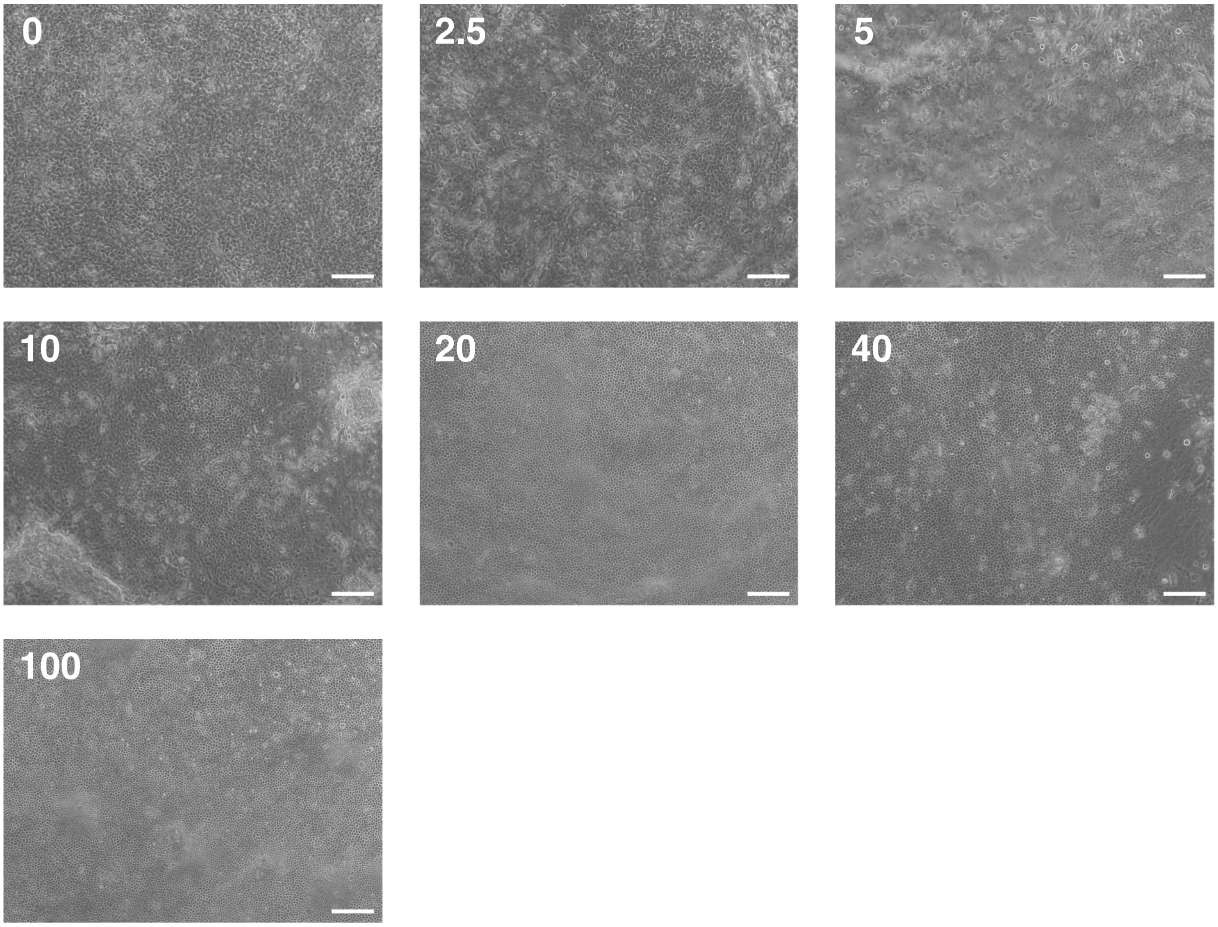

Supplementary Figure S6. LECs cultured with Y-27632 and different concentration of KGF.

(a) LEC proliferation ability and KGF concentration. Fluorescence intensity is proportional to the number of viable cells. **(b)** Phase contrast micrograph of LECs cultured with Y-27632 and different concentration of KGF (represented at top left) at day 19. Highly dense and uniform LECs are observed when cultured with 20 ng/ml KGF and Y-27632. Scale bars are 200μm.

Supplementary Figure S7

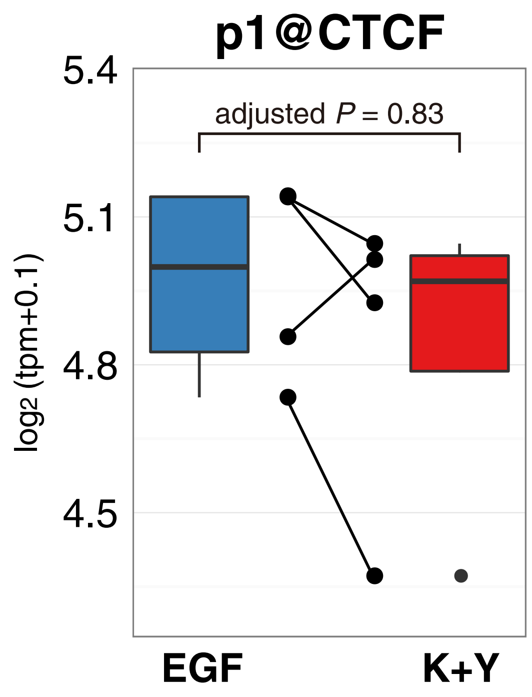

Supplementary Figure S7. Expression changes of the main promoter of *CTCF*, p1@CTCF in LECs cultured in the two conditions.

K+Y: KGF and Y-27632.

Supplementary Table S1. List of 877 promoters highly expressed in K+Y-treated LECs.

See the corresponding Excel file. Transcription factors (22 promoters) are highlighted in bold characters. Promoters are sorted by statistical significance (adjusted *P*-value). Specificity is based on the microarray data GSE5543.

Supplementary Table S2. List of 859 promoters highly expressed in EGF-treated LECs.

See the corresponding Excel file. Promoters are sorted by statistical significance (adjusted *P*-value). Specificity is based on the microarray data GSE5543.

Supplementary Table S3. Expression levels of the *miR-184* promoter, p1@MIR184 in the samples registered in the FANTOM5 database.

p1@MIR184	
**Sample**	**Expression level (tpm)**
**Lens Epithelial Cells**, donor2.	5.56
**Ciliary Epithelial Cells**, donor3.	1.49
**Ciliary Epithelial Cells**, donor1.	1.42
**eye**, fetal, donor1.	1.36
**Iris Pigment Epithelial Cells**, donor1.	0.86
**Ciliary Epithelial Cells**, donor2.	0.67
testicular germ cell embryonal carcinoma cell line:NEC14.	0.53
pineal gland, adult, donor10252.	0.34
**Retinal Pigment Epithelial Cells**, donor3.	0.33
small cell lung carcinoma cell line:NCI-H82.	0.11

The top 10 samples are presented.
Ocular tissues are shown in bold.

tpm: tags per uniquely mapped million tags.

Supplementary Table S4. Expression levels of the alternative *PAX6* promoters in the samples registered in the FANTOM5 database.

p1@PAX6	
**Sample**	**Expression level (tpm)**
cerebellum - adult, donor10196.	212.09
cerebellum, adult, donor10252.	123.52
Neural stem cells, donor1.	80.55
carcinosarcoma cell line:JHUCS-1.	75.01
argyrophil small cell carcinoma cell line:TC-YIK.	66.01
gastric cancer cell line:AZ521.	57.84
small cell lung carcinoma cell line:DMS 144.	55.77
cerebellum, adult, pool1.	54.97
cord blood derived cell line:COBL-a 24h infection(-C).	53.37
**Ciliary Epithelial Cells**, donor1.	52.09
**p2@PAX6**	
**Sample**	**Expression level (tpm)**
small cell lung carcinoma cell line:DMS 144.	140.33
cerebellum, adult, donor10252.	119.65
cerebellum - adult, donor10196.	68.66
hippocampus, adult, donor10252.	37.59
caudate nucleus - adult, donor10196.	34.54
Neural stem cells, donor2.	29.93
middle temporal gyrus, donor10252.	29.86
medial temporal gyrus, adult, donor10252.	29.67
Astrocyte - cerebral cortex, donor1.	28.05
occipital cortex, adult, donor10252.	26.82

p3@PAX6	
**Sample**	**Expression level (tpm)**
argyrophil small cell carcinoma cell line:TC-YIK.	95.99
**Lens Epithelial Cells**, donor2.	81.96
**eye**, fetal, donor1.	80.45
**Ciliary Epithelial Cells**, donor1.	79.79
small cell lung carcinoma cell line:DMS 144.	57.2
**Ciliary Epithelial Cells**, donor2.	51.44
**Ciliary Epithelial Cells**, donor3.	50.24
**Corneal Epithelial Cells**, donor3.	38.56
**Iris Pigment Epithelial Cells**, donor1.	34.31
Astrocyte - cerebral cortex, donor1.	30.34
**p9@PAX6**	
**Sample**	**Expression level (tpm)**
argyrophil small cell carcinoma cell line:TC-YIK.	13.35
**Lens Epithelial Cells**, donor2.	11.36
**eye**, fetal, donor1.	11.3
cerebellum - adult, donor10196.	7.63
occipital lobe, fetal, donor1.	6.3
Astrocyte - cerebral cortex, donor1.	5.72
**Ciliary Epithelial Cells**, donor1.	5.29
**Ciliary Epithelial Cells**, donor3.	4.13
**Iris Pigment Epithelial Cells**, donor1.	4.12
**Ciliary Epithelial Cells**, donor2.	3.99

The top 10 samples are presented.
Ocular tissues are shown in bold.

tpm: tags per uniquely mapped million tags.

Supplementary Table S5. Donor information, RNA integrity number (RIN), and summary of sequence statistics for the CAGE analysis.

Sample name	Age	Sex	Days of culture	RIN	Total number of reads	Total number of mapped reads	Mapping rate (%)
K+Y_1	62	Male	19	9.5	4,196,890	3,383,622	80.6
EGF_1	62	Male	19	9.5	8,317,942	7,112,053	85.5
K+Y_2	53	Female	19	9.5	8,409,102	6,964,907	82.8
EGF_2	53	Female	19	9.6	9,612,868	8,279,296	86.1
K+Y_3	58	Male	17	9.7	4,546,196	3,495,864	76.9
EGF_3	58	Male	17	9.6	5,305,605	4,444,418	83.8
K+Y_4	69	Female	17	9.5	2,527,049	2,149,860	85.1
EGF_4	69	Female	17	9.5	17,524,657	14,972,941	85.4

Supplementary Table S6. List of probes used for the TaqMan Gene Expression Assay.

Gene	Taqman probe ID
GAPDH	Hs99999905_m1
KRT3	Hs00365080_m1
KRT12	Hs00165015_m1
CLU	Hs00971656_m1
ALDH3A1	Hs00964880_m1
ANGPTL7	Hs00221727_m1
TKT	Hs01115545_m1

Supplementary Table S7. List of probes used for the TaqMan MicroRNA Assay.

Gene	Assay ID
U6 snRNA	001973
miR-184	000485

Supplementary Table S8. List of primers designed for the SYBR assay.

	Forward primer	Reverse primer
GAPDH	AGCCTCCCGCTTCGCTCTCT	CCAGGCGCCCAATACGACCA
PAX6-short	TGAGCCATCACCAATCAGCA	TAAAATGGGCTGTCAGCGGC
PAX6-long	ACATTTAAACTCTGGGGCAGGT	TCAGATTCCTGGGAGCGGAG
